# Commentary: Utility-free heuristic models of two-option choice can mimic predictions of utility-stage models under many conditions

**DOI:** 10.3389/fnins.2015.00188

**Published:** 2015-05-27

**Authors:** Camillo Padoa-Schioppa

**Affiliations:** Departments of Anatomy and Neurobiology, Economics, and Biomedical Engineering, Washington University in St. LouisSt. Louis, MO, USA

**Keywords:** decision making, value comparison, heuristics, dimensional prioritization, value correlate, utility, subjective value, neuroeconomics

Many neuroeconomic studies in the past 10 years have reported neural signals encoding the subjective value (or utility) of offered and chosen goods (Padoa-Schioppa, 2011; Bartra et al., 2013; Clithero and Rangel, 2014; but see O'Doherty, 2014). The precise mechanisms through which values are compared to make a decision remain unclear and are matter of current research. However, the fact that neurons in the orbitofrontal and ventromedial prefrontal cortices encode the subjective value of offered and chosen goods, taken together with the fact that lesions to these same areas selectively disrupt economic decisions (Camille et al., 2011), strongly suggests that economic choices are ultimately based on these value signals (Kable and Glimcher, 2009; Rangel and Hare, 2010; Padoa-Schioppa, 2011). These results are generally viewed as a significant breakthrough compared to standard and behavioral economic theories, where subjective value (or utility) enters as an “as if” concept. In a recent paper, Piantadosi and Hayden (2015) challenge this understanding.

Goods available for choice can generally vary on multiple dimensions (or attributes), and by definition subjective values integrate all the dimensions relevant to the decision. The authors first examine the specific case in which choices are made between two options that depend only on quantity and probability (two parametric dimensions). Building on an argument originally put forth by Tversky (1969), they show that decisions based on an integrated utility (algorithm 1) cannot be distinguished from decisions based on a utility-free heuristic (algorithm 2). According to this second algorithm, subjects would first identify the attribute with highest variance and then choose according to that attribute alone (dimensional prioritization). This heuristic does not include the computation of any utility. The authors go on to claim that the same argument applies very broadly to binary decisions between goods that vary on two or more dimensions, provided that the dimensions are decomposable into additive functions. In the last part of the paper, they claim that similar arguments apply to neural data and that neural signals previously found to encode subjective value “*may arise artifactually from utility-free heuristic processes.*”

The paper is interesting for it highlights one of the challenges in linking a behavioral choice or a neural signal to the computation of subjective values. Importantly, if the algorithm of Piantadosi and Hayden applied as broadly as the authors claim it does, a pillar of neuroeconomics would find itself on shaky ground. However, upon a closer examination it appears clear that the authors greatly overstated their case, and that the domain of decisions to which their utility-free heuristic applies is actually rather limited. In particular, algorithm 2 fails whenever choices are made between qualitatively different (incommensurable) goods. After discussing the specific case in which options depend only on quantity and probability, the authors write:

“Moreover, there is nothing special about the fact that these choices involve risk. For example, in a well-known study, subjects choose between two amounts of juice that differ in flavor and quantity (Padoa-Schioppa and Assad, 2006). […] It is plausible to assume that in [that study], the utility of each option may be a product of its scalar values along the two dimensions. If so, it is straightforward to create a utility-free algorithm that makes the same choices as the choice model using the same principles”

This statement is actually incorrect. Consider a subject choosing between different quantities of apple juice and orange juice. Importantly, flavor is a subjective and complex sensation that cannot be reduced to the sum of simple components (Small, 2012; Spence, 2015). Thus different flavors are effectively incommensurable commodities. The statement is incorrect because there is no parametric dimension along which two flavors can be assigned a scalar value. Put in a different way, consider decisions between option 1 = [apple flavor, quantity 1] and option 2 = [orange flavor, quantity 2]. If the first dimension was probability instead of flavor, one could say that probability 1 is larger or smaller than probability 2. But one cannot say that apple flavor is larger or smaller than orange flavor: such statement would be meaningless. As a consequence, one cannot compute the variance in the flavor dimension, as required by algorithm 2. The only way to compare apple flavor and orange flavor is to compute their subjective value, which is precisely what Piantadosi and Hayden argue against. Thus, algorithm 2 cannot account for choices as simple as that between an apple and an orange. More generally, algorithm 2 fails whenever choices are made between incommensurable goods.

Algorithm 2 also fails in simple cases where goods vary only on parametric dimensions. For example, consider the choice between two lottery tickets. Each ticket costs C_i_ dollars and pays R_i_ dollars with probability P_i_ (*i* = 1, 2). Lets consider the simple case of linear value function and risk indifference. In the integrated value framework, choices are made according to the utility function *U* = P R – C. This utility function cannot be decomposed into additive functions. Thus, the argument of Piantadosi and Hayden falls.

Note that choices between incommensurable goods are not esoteric cases—they are ubiquitous in the life of humans and other animals. For example, people make such choices every time they exchange money for another commodity—work, food, sex, etc. Likewise, choices where attributes include quantity, a multiplicative dimension such as probability or time, and an additive dimension such as cost are very common. The vast majority of daily economic decisions seem to fall in one of those classes, and algorithm 2 cannot account for any of them.

The issues raised above are also relevant to the neuroscience implications discussed in the paper. Much of the evidence for neural signals encoding the subjective value of offered and chosen goods comes from studies in which choices were made between qualitatively different goods. These include the juice choice study discussed above and many other (Padoa-Schioppa and Assad, 2006; Plassmann et al., 2007; Valentin et al., 2007; FitzGerald et al., 2009; Raghuraman and Padoa-Schioppa, 2014). Contrary to the claims of Piantadosi and Hayden, the results presented in their paper *do not* provide any indication that neural value signals found in those studies “*may arise artifactually from utility-free heuristic processes.*” Indeed, Piantadosi and Hayden *have not* “*identified a potential confound to neural correlates of utility*” in those studies.

## Conflict of interest statement

The author declares that the research was conducted in the absence of any commercial or financial relationships that could be construed as a potential conflict of interest.
